# Early Breast Milk Volumes and Response to Galactogogue Treatment

**DOI:** 10.3390/children9071042

**Published:** 2022-07-13

**Authors:** Elizabeth V. Asztalos, Alex Kiss

**Affiliations:** 1 Sunnybrook Health Sciences Centre, Department of Paediatrics, University of Toronto, Toronto, ON M4N 3M5, Canada; 2 Sunnybrook Research Institute, Sunnybrook Health Sciences Centre, University of Toronto, Toronto, ON M4N 3M5, Canada; alex.kiss@ices.on.ca

**Keywords:** early breast milk volumes, early breast milk production, galactagogues, mothers of preterm infants

## Abstract

The aim of this study was to evaluate the effect of galactogogue management in mothers of very preterm infants with varying breast milk volumes prior to initiating this treatment. Data were utilized from 90 women who participated in a trial employing domperidone. Three groups were formed according to their breast milk volumes (based on their infants’ birth weight) at the time of randomization and study entry to the trial protocol: (1) ≤100 mL/kg/d; (2) 101–200 mL/kg/d; and (3) ≥201 mL/kg/d. Breast milk volumes were evaluated at the 14- and 28-day study treatment periods. All three groups showed a significant volume increase and volume percent increase both at the 14-day measure and also the 28-day measure. Mothers who started in the two lower volume groups showed the greatest % volume change overall, with 356.2% in the ≤100 mL/kg/d and 106.1% in the 101–200 mL/kg/d groups, compared to those mothers in the higher group of ≥201 mL/kg/d, showing a change of 45.2%, where *p* = 0.001. Mothers producing varying low volumes were able to demonstrate an effect from the use of domperidone and increase their volumes as much as three-hundred-fold over 14- and 28-day study periods. However, those mothers whose volumes were ≤100 mL/kg/d continued to maintain low absolute milk volumes, putting these mothers at ongoing risk of ceasing lactation.

## 1. Introduction

The very preterm/very low birthweight infant (under 30 weeks gestation or under 1500 g at birth) is faced with a significant array of morbidities following an early birth. These morbidities can include late-onset sepsis, necrotizing enterocolitis, bronchopulmonary dysplasia, retinopathy of prematurity, and significant white matter brain injury [1,2,3,4,5]. All of these morbidities contribute, one way or another, to developmental delays of varying severity [6,7]. The active ingredients found in human milk, and, in particular, the mother’s milk, are protective, function as a substrate for the development of organs, and play a major role in reducing serious morbidities [6,7]. This has led to the recommendation that the very preterm infant be given human milk as their primary source of nutrition [8]. Given the significant benefits associated with the mother’s own milk, mothers of very preterm infants have been encouraged to provide their own pumped breast milk for their infants even in the presence of readily available donor breast milk. Studies have shown the importance of establishing an adequate milk supply in the early postpartum period in an effort to maintain volumes in sufficient amounts to meet the nutritional needs of their infants during their lengthy stay in hospital, which can range anywhere from 10 to 16 weeks or even longer [9]. There are known interventions that function to assist mothers of very preterm infants with breast milk production [7]; however, responses to these interventions can be variable. Many mothers of very preterm infants, for a variety of reasons such as illness, stress, and other factors related to preterm birth, may struggle to provide breast milk exclusively to their children. When these supportive interventions are unsuccessful, galactogogues are often considered in the presence of these ongoing challenges in breast milk production. Their specific mode of action is to augment milk production by exerting effects either through the use of oxytocin or prolactin [10]. One of the most commonly used galactogogues is domperidone, which is a dopamine antagonist that exerts an effect by increasing the serum prolactin levels, which then help to stimulate milk production in the breasts [10].

A recent trial, ***EMPOWER***, was able to demonstrate that the administration of domperidone, initiated within the first 21 days after delivery, would lead to a modest increase in milk volume [11,12]. Although the results of the trial supported the use of a galactogogue, in this case, domperidone, clarity regarding what to expect from a galactogogue is still needed. Hence, the aim of this study was to evaluate the response pattern to a galactogogue in mothers with varying low breast milk volumes prior to initiating this treatment.

## 2. Materials and Methods

For the purpose of this study, data from the ***EMPOWER*** trial were utilized; the ***EMPOWER*** trial has previously been described in detail [11,12]. To summarize, mothers ranging from 8 to 21 days postpartum whose infants were born between 23^0/7^ and 29^6/7^ gestation were approached to participate in a trial. Initially, the mothers needed to pump an average of ≤150 mL/kg/d for 72 h prior to entry; this was changed to ≤250 mL/kg/d one year into the study, as this criterion was felt to be too restrictive. The volume was based on their infant’s birth weight in kilograms (kg); in the case of twins, the volume was based on the larger twin’s birthweight. In the trial, the study treatment was given three times/day at a dose of 10 mg for a total of 28 days. In the first 14-day period, the mothers were randomized to either receive the active study drug or a comparable placebo. All the mothers received domperidone between days 15 and 28 of the study treatment.

For the purpose of this study, three groups of mothers were formed based on their breast milk volumes (mL/kg/day, based on the infants’ birth weight) at the time of randomization and study entry: (1) ≤100 mL/kg/d; (2) 101–200 mL/kg/d; and (3) ≥201–250 mL/kg/d. These mothers were grouped in these volume groups for ease of analysis; the grouping volumes had no bearing on any known physiologic factors but were similar to volumes associated with a low chance of successful breast milk production [13].

The primary outcome of this study was the mean percent absolute volume change from start to day 14 and from day 15 to day 28 of the study treatment period.

The secondary outcome measures of the study were: (i) the proportion of mothers who achieved a 50% increase in breast milk volume by day 28 of the study treatment period and (ii) the number of mothers who achieved a milk volume of 500 mL/d on days 14 and 28 of the study treatment period.

The analysis was carried out using SAS Version 9.3 (SAS Institute, Cary, NC, USA). Descriptive statistics were calculated for all variables of interest. Continuous measures were summarized using means and standard deviations, whereas categorical measures were summarized using counts and percentages. Chi-square and Fisher’s exact tests were used to compare categorical variables between groups, whereas milk volume was compared between groups using an analysis of variance (ANOVA).

The primary and secondary outcomes were assessed between groups, with pairwise comparisons based on logistic regression models, controlling for the use of domperidone, as outlined in the trial description. Adjusted odds ratios were presented along with their associated 95% confidence intervals and *p*-values. The changes in milk volume, as a continuous measure, were also compared between groups. This was carried out using a linear mixed model with patient as a random effect. The model components were group (≤100 mL, 101–200 mL, ≥201 mL), time (0, 14 and 28 days), and a group by time interaction term. Finally, a post-hoc analysis utilizing the non-parametric Wilcoxon rank sum test was conducted within each milk volume study group to evaluate any difference that may have existed in the first 14 days of the study treatment.

## 3. Results

Data for a total of 90 mothers were included in this analysis. Of the 90 mothers enrolled in the study, 37 gave birth to infants from 23 to 26 completed weeks of gestation, and 53 gave birth to infants from 27 to 29 completed weeks of gestation. Table 1 outlines the characteristics of the mothers in the three groups of study. The three groups of mothers were similar in terms of their rates of cesarean section, twin pregnancies, and exposure to antenatal corticosteroids.

Table 2, which outlines the primary outcome, shows the volumes achieved and the percent volume changes seen in each starting volume group. All three groups showed a significant volume increase and volume percent increase at both the 14-day measure and the 28-day measure. For all three groups, the greatest increase was seen in the first 2-week block. In the original EMPOWER trial, the mothers were randomized to enter the first 2 weeks of the study treatment as either a domperidone or a placebo participant, and only in days 15–28 of the study treatment did all mothers receive domperidone. Because half of the mothers received a placebo from days 1 to 14, we elected to observe the volume changes in each group of mothers to identify the effects of not only domperidone but also supportive measures. Table 3 demonstrates that the mothers who received the placebo study treatment demonstrated an increase in breast milk volume, but not as much as those who received the domperidone.

Table 4 shows the proportion of mothers in the three volume groups who achieved a 50% increase in expressed milk volume on day 28 of the study treatment. All three groups were similar in their proportions, 72% vs. 60% vs. 58%, where *p* = 0.45.

Finally, Table 5 shows the proportion of mothers who achieved the target volume of 500 mL/d on days 14 and 28. The highest volume group (≥201 mL/kg/d) was 4.7 and 6.3 times more likely to achieve this target volume when compared to the ≤100mL/kg/d and 101–200 mL/kg/d groups, respectively.

The results of the linear mixed model to assess change in milk volume as a continuous measure over time between groups produced a non-significant *p*-value for the group by time interaction term (*p* = 0.69). Groups 2 and 3 were found to have significantly higher milk volumes across all time points than group 1 (both *p* < 0.0001), with the volumes of group 3 also being significantly higher than those of group 2 (*p* = 0.008).

## 4. Discussion

The aim of this study was to evaluate the response patterns to a galactogogue among mothers with varying low breast milk volumes prior to initiating this treatment. Overall, we saw that the mothers demonstrated an increase in absolute milk volume, regardless of what their initial volume was. Of note, the greatest % volume change was found among those mothers who started off with the lowest initial volume (≤100 mL/kg/d based on their infant’s birthweight). All three groups showed that this could be maintained for the total of 28 days. This is in keeping with the findings of other studies that have evaluated the use of pharmacologic strategies—in particular, domperidone—to aid in helping mothers with increasing their breast milk supply [14,15].

Although many factors have been associated with breastfeeding challenges in mothers of preterm infants [16,17,18], the establishment of breast milk production in the first week postpartum plays a major role [13,19,20]. A volume of <1700 mL/week, or an average of 250 mL/day, has been cited as a cut-off for a mother’s chance of achieving an adequate production of milk by week 6 postpartum and after [13]. However, within that volume cut-off of 1700 mL/week, it was not evident whether mothers would be able to respond to various interventions, including galactogogue treatments, and what the magnitude of their response would be. The mothers in the lowest volume group, in fact, had the highest mean % volume change for the first 14 days of over 350%. However, despite this % volume change, it is important to note that this group still had quite low absolute milk volumes at the two specified timepoints of measure in the study: days 14 and 28. Volumes still remained significantly lower in this group when compared to their counterparts. It suggests that this group of mothers, those who produced ≤100 mL/kg/d by the end of the first post-partum week, remain at high risk of continued production of low volume. This may be due to them having a lower number of receptors for prolactin to aid in stimulating milk production. If receptors remain at a lower level, the use of a galactogogue, whose primary mode of action is to increase prolactin, will lead to an ineffective response. This is a group of mothers who will need added support and encouragement to continue to pump to produce whatever volumes of breast milk possible for them; however, this study notes that they may never be able to fully provide breast milk for their infants and will need to rely on donor milk and/or formula for nutritional support [9,13]. However, having more mothers achieve a gain in breast milk volume, regardless of these low starting volumes, means more of the mother’s own milk will be available to a preterm infant. This is of critical importance in this population of preterm mothers and their infants, as it has been seen that an increase in the mother’s own milk by 1 mL/kg/d is associated with a 1-point higher score on neurocognitive assessment results of infants by 2+ years corrected age [7].

The volume of 500 mL/day has also been cited as a goal to meet the nutritional needs of the preterm infant [13]. We saw in this analysis that only in the higher volume group (≥201 mL/kg/d) were mothers able to produce adequate volumes in a range close to 500 mL/day; yet, only 42% of the mothers in this group were able to achieve this volume by the end of the 28-day study period. However, again, one recognizes that the number of mothers in this group was small and, therefore, there is limited power to draw meaningful conclusions from this observation.

There are numerous maternal and obstetric factors which can modify how lactogenesis may occur in mothers of preterm infants [16]. Antenatal corticosteroids have been found to have an effect on reduced breast milk volumes, in particular in women who have delivered at the very preterm gestational age [21]. Similarly, differences in various co-morbidities have been reported to have an effect on this [16]. In this analysis, the use of antenatal corticosteroids was similar among the mothers of the three groups, and we did not find an effect from co-morbidities in milk volume production between the three groups.

The ***EMPOWER*** trial used different strategies to determine the response patterns of galactogogues in a high-risk population of mothers [11,12]. The gains seen by day 14 were seen across all of the three groups. Because we know that almost half of the mothers in the study groups were receiving a placebo in the first 14 days, this also suggests that non-pharmacologic strategies remain critically important for supporting breast milk production in these early postpartum weeks and, in fact, can help to augment breast milk production almost to the same level as that of mothers who receive a galactogogue. Mothers were encouraged to pump a minimum of 6–8 times/24 h period. Centres worked diligently with the mothers to provide kangaroo care and all had strategies in place to help mothers initiate and support breast milk expression. All of these strategies are known to play an instrumental role in aiding high-risk mothers in their breast milk expression and lactogenesis in the early postpartum weeks [22,23].

There are strengths and limitations to this study. The findings of this study are based on a population of mothers who were recruited for the purposes of an already published, randomized, controlled trial. Our three groups of mothers who participated in the analysis were similar in characteristics, and we did not see any effects from any potential confounding variables, despite concerns regarding it potentially being underpowered. An important limitation to this study is that the mothers in the original trial were on differing treatment schedules: Group A took 10 mg of domperidone orally three times daily for 28 days; Group B took 10 mg of a placebo orally three times daily for 14 days followed by 10 mg of domperidone orally three times daily for 14 days. For this current study, we attempted to assess our outcomes with pairwise comparisons to control for the effects of the varying domperidone approaches as noted in the trial; however, we do need to acknowledge that this may be a limitation in drawing strong conclusions from our study. As previously noted, some of our analyses did not reach statistical significance as well as having wide confidence intervals, suggesting that our sample size may have been too small to answer some of our questions. However, our sample size was adequate to achieve our primary aim.

A final point to address relates to the risks and benefits of any treatment approach that a clinician may consider for a patient. Over the past decade, concerns have risen related to the safety of domperidone. Domperidone has been associated with prolonged Q-Tc interval, a higher risk of cardiac arrhythmias, and sudden death [24,25,26]. The relevance of these cardiac findings when domperidone is used for lactation support is not clear. However, regulatory agencies have made recommendations regarding how lactating women should use domperidone [27,28]. These concerns do need to be taken into account by the clinician when considering domperidone as a galactogogue support.

## 5. Conclusions

In this study, we saw that mothers with varying milk volumes were able to increase their volumes as much as three-hundred-fold over 14- and 28-day study periods. However, those mothers whose volumes were ≤100 mL/kg/d at the start continued to maintain low absolute milk volumes, putting these mothers at ongoing risk of ceasing lactation. Mothers of very preterm infants should be identified by the end of the first postpartum week for any evidence of low milk production. These identified mothers need intensive support and encouragement to continue to produce milk for their infants. A very focused approach that is aimed at supporting whatever breast milk production these mothers are able to achieve should be in place and should include pumping support to maintain the autocrine function of the breast and the development of prolactin receptors, with the consideration of the use of a galactogogue as soon as the end of the first week after giving birth to help optimize breast milk production [22,23].

## Figures and Tables

**Table 1:** Baseline characteristics of mothers.

	Milk Volume Level			
Characteristics	≤100 mL/kg/d N = 46	101–200 mL/kg/d N = 25	≥201 mL/kg/d N = 19	*p*-Value *
Maternal age (year) mean (SD) (min, max)	31.0 (6.6) (19.7, 44.6)	32.4 (4.7) (19.4, 38.4)	30.9 (5.7) (21.3, 40.6)	*p* = 0.60
Self-declared ethnicity, n (%) Caucasian Black Asian Aboriginal/other	28 (60.9) 6 (13.0) 8 (17.4) 4 (8.7)	16 (64.0%) 2 (8.0%) 6 (24.0%) 1 (4.0%)	15 (78.9%) 2 (10.5%) 2 (10.5%) 0 (0%)	*p* = 0.53
Primigravida, n (%)	17 (36.9)	9 (36.0%)	5 (26.3%)	*p* = 0.74
Co-Morbidities during pregnancy, n (%) Hypertension (gestational/chronic) Diabetes (gestational/Type I and II) Preterm labour Chorioamnionitis Antepartum haemorrhage Other	26 (56.5) 7 3 7 2 6 9	12 (48.0%) 3 3 3 0 3 6	8 (42.1%) 2 2 2 1 1 3	*p* = 0.54
Antenatal corticosteroids, n (%)	38 (82.6)	21 (84.0)	17 (89.5)	*p* = 0.86
Cesarean Section, n (%)	27 (58.7)	12 (48.0%)	9 (47.4%)	*p* = 0.58
Twin Pregnancy, n (%)	7 (15.2)	3 (12.0%)	3 (15.8%)	*p* = 1.00

* *p*-value for maternal age was based on ANOVA, and for others were based on chi square test or Fisher’s exact test. SD = standard deviation.

**Table 2:** Milk volumes at study time points day 0, day 14, and day 28.

	Milk Volume Level			
Milk Volume Parameters	≤100 mL/kg/d ^†^ N = 45 (1)	101–200 mL/kg/d ^†^ N = 25 (2)	≥201 mL/kg/d ^†^ N = 19 (3)	*p*-Value *
Baseline volume (mL) prior to starting study treatment (SD)	42.2 (29.3)	149.9 (37.7)	260.8 (54.6)	*p* < 0.0001
Volume on study treatment day 14, (mL) mean (SD)	155.9 (160.3)	303.0 (147.8)	375.8 (154.6)	*p* < 0.0001
Volume on study treatment day 28, (mL) mean (SD)	199.6 (203.1)	349.1 (163.5)	454.3 (212.9)	*p* < 0.0001
Mean % volume change day 0 to day 14 (%) (SD)	356.2 (465.8)	106.1 (99.2)	45.2 (62.5)	*p* = 0.001
Mean % volume change day 15 to day 28 (%) (SD)	42.0 (66.7)	23.7 (38.6)	29.2 (63.0)	*p* = 0.48

* *p*-values based on ANOVA. mL = milliliters ^†^ based on infant’s birthweight (kg). SD = standard deviation.

**Table 3:** Milk volumes at study time points day 0 and day 14 according to *EMPOWER* study allocation.

Milk Volume Parameter	≤100 mL/kg/d N = 46			101–200 mL/kg/d N = 25			≥201 mL/kg/d N = 19		
	Domperidone N = 23	Placebo N = 23		Domperidone N = 13	Placebo N = 12		Domperidone N = 9	Placebo N = 10	
Baseline volume (mL) prior to starting study treatment (SD)	44.48 (33.68)	39.97 (24.81)	*p*	158.95 (41.30)	140.19 (32.13)	*p*	263.78 (56.37)	258.17 (55.81)	*p*
Volume on study treatment day 14 (mL) mean (SD)	142.17 (114.48)	171.75 (202.78)	0.73	350.46 (123.72)	246.82 (81.77)	0.20	468.67 (106.48)	283.00 (141.58)	0.006
Mean % volume change day 0 to day 14 (%) (SD)	400.64 (571.10)	304.80 (310.53)	0.99	123.72 (117.58)	85.33 (71.77)	0.41	81.50 (47.24)	8.95 (55.85)	0.01

*p* = *p* values (based on non-parametric Wilcoxon rank sum test).

**Table 4:** Secondary outcome: number of women achieving 50% increase in milk volume by day 28.

	Milk Volume Level				
	≤100 mL/kg/d (n = 46) (1)	101–200 mL/kg/d (n = 25) (2)	≥201 mL/kg/d (n = 19) (3)	Adjusted Odds Ratio (95% Confidence Interval)	*p*-Value *
Mothers who achieved 50% increase in milk volume on day 28, n (%)	33 (71.7)	15 (60.0)	11 (57.9)	2 vs. 1 0.59 (0.21, 1.66) 3 vs. 1 0.54 (0.17, 1.68) 3 vs. 2 0.91 (0.26, 3.18)	*p* = 0.88 *p* = 0.32 *p* = 0.29

* *p*-values were obtained from pairwise comparisons based on logistic regressions controlling for the use of domperidone.

**Table 5:** Secondary outcome: proportion of mothers who achieved 500 mL/day by days 14 and 28 of study period.

	Milk Volume Level				
Milk Volume Parameters	≤100 mL/kg/d N = 46 (1)	101–200 mL/kg/d N = 25 (2)	≥201 mL/kg/d N = 19 (3)	Adjusted Odds Ratio (95% Confidence Interval)	*p*-Value *
Mothers who achieved a volume of 500 mL/d by day 14, n (%)	2 (4.6%)	3 (12.5%)	4 (22.2%)	2 vs. 1 2.94 (0.45, 19.06) 3 vs. 1 5.81 (0.92, 36.65) 3 vs. 2 1.98 (0.35, 11.07)	*p* = 0.26 *p* = 0.06 *p* = 0.44
Mothers who achieved a volume of 500 mL/d by day 28, n (%)	6 (13.9%)	4 (18.2%)	8 (50.0%)	2 vs. 1 1.36 (0.34, 5.45) 3 vs. 1 6.33 (1.64, 24.48) 3 vs. 2 4.66 (1.00, 21.62)	*p* = 0.67 *p* = 0.01 *p* = 0.05

* *p*-values were obtained from pairwise comparisons based on logistic regressions controlling for the use of domperidone. * Note: 5 missing cases in milk volume at day 14 and 9 missing cases in milk volume at day 28.

## Data Availability

Data supporting this project can be found at Sunnybrook Research Institute, Sunnybrook Health Sciences Centre, Toronto, ON, and are available from Elizabeth Asztalos on request.

## References

[1] Maffei D., Schanler R.J. (2017). Human milk is the feeding strategy to prevent necrotizing enterocolitis!. Semin Perinatol..

[2] Abrams S.A., Schanler R.J., Lee M.L., Rechtman D.J. (2014). Greater mortality and morbidity in extremely preterm infants fed a diet containing cow milk protein products. Breastfeed Med..

[3] Schanler R.J. (2007). Mother’s own milk, donor human milk, and preterm formulas in the feeding of extremely premature infants. J. Pediatr. Gastroenterol. Nutr..

[4] Morales Y., Schanler R.J. (2007). Human milk and clinical outcomes in VLBW infants: How compelling is the evidence of benefit?. Semin. Perinatol..

[5] Porcelli P.J., Weaver R.G. (2010). The influence of early postnatal nutrition on retinopathy of prematurity in extremely low birth weight infants. Early Hum. Dev..

[6] Vohr B.R., Poindexter B.B., Dusick A.M., McKinley L.T., Wright L.L., Langer J.C., Poole W.K., for the NICHD Neonatal Research Network (2006). Beneficial effects of breast milk in the neonatal intensive care unit on the developmental outcome of extremely low birth weight infants at 18 months of age. Pediatrics.

[7] Lechner B.E., Vohr B.R. (2017). Neurodevelopmental outcomes of preterm infants fed human milk: A systematic review. Clin Perinatol..

[8] Moro G.E., Arslanoglu S., Bertino E., Corvaglia L., Montirosso R., Picaud J.C., Polberger S., Schanler R.J., Steel C., van Goudoever J. (2015). American Academy of Pediatrics; European Society for Pediatric Gastroenterology, Hepatology, and Nutrition. Human milk in feeding premature infants: Consensus statement. J. Pediatr. Gastroenterol. Nutr..

[9] Hill P.D., Aldag J.C., Chatterton R.T., Zinaman M.J. (2005). Comparison of milk production between mothers of preterm and term mothers: The first six weeks after birth. J. Hum. Lact..

[10] Gabay M.P. (2002). Galactogogues: Medications that induce lactation. J. Hum. Lact..

[11] Asztalos E.V., Campbell-Yeo M., daSilva O.P., Kiss A., Knoppert D.C., Ito S. (2012). Enhancing breast milk production with Domperidone in mothers of preterm neonates (EMPOWER trial). BMC Pregnancy Childbirth.

[12] Asztalos E.V., Campbell-Yeo M., da Silva O.P., Ito S., Kiss A., Knoppert D., EMPOWER Study Collaborative Group (2017). Enhancing Human Milk Production With Domperidone in Mothers of Preterm Infants. J. Hum. Lact..

[13] Hill P.D., Aldag J.C., Chattertoon R.T. (1999). Effects of pumping style on milk production in mothers of non-nursing preterm infants. J. Hum. Lact..

[14] Grzeskowiak L.E., Smithers L.G., Amir L.H., Grivell R.M. (2018). Domperidone for increasing breast milk volume in mothers expressing breast milk for their preterm infants: A systematic review and meta-analysis. BJOG.

[15] Shen Q., Khan K.S., Du M.-C., Du W.-W., Ouyang Y.-Q. (2021). Efficacy and Safety of Domperidone and Metoclopramide in Breastfeeding: A Systematic Review and Meta-Analysis. Breastfeed. Med..

[16] Sievers Haase S., Oldigs H.-D., Schaub J. (2003). The impact of peripartum factors on the onset and duration of lactation. Biol. Neonate.

[17] Zachariassen G., Faerk J., Grytter C., Esberg B., Juvonen P., Halken S. (2010). Factors associated with successful establishment of breastfeeding in very preterm infants. Acta Paediatr..

[18] Maastrup R., Hansen B.M., Kronborg H., Bojesen S.N., Hallum K., Frandsen A., Kyhnaeb A., Svarer I., Hallström I. (2014). Factors associated with exclusive breastfeeding of preterm infants. Results from a prospective national cohort study. PLoS ONE.

[19] Hill P.D., Aldag J.C. (2005). Milk volume on day 4 and income predictive of lactation adequacy at 6 weeks of mothers of nonnursing preterm infants. J. Perinat. Neonatal Nurs..

[20] Hill P.D., Aldag J.C., Zinaman M., Chatterton R.T. (2007). Predictors of preterm infant feeding methods and perceived insufficient milk supply at week 12 postpartum. J. Hum. Lact..

[21] Henderson J.J., Hartmann P.E., Newnham J.P., Simmer K. (2008). Effect of preterm birth and antenatal corticosteroid treatment on lactogenesis II in women. Pediatrics.

[22] Renfrew M.J., Dyson L., McCormick F., Misso K., Stenhouse E., King S.E., Williams A.F. (2010). Breastfeeding promotion for infants in neonatal units: A systematic review. Child Care Health Dev..

[23] Parker L.A., Sullivan S., Krueger C., Kelechi T., Mueller M. (2013). Strategies to increase milk volume in mother of VLBW. MCN Am. J. Matern. Child Nurs..

[24] Grzeskowiak L. (2014). Use of domperidone to increase breast milk supply: Are women really dying to breastfeed?. J. Hum. Lact..

[25] Sewell C.A., Chang C.Y., Chehab M.M., Nguyen C.P. (2017). Domperidone for Lactation: What Health Care Providers Need to Know. Obstet. Gynecol..

[26] Buffery P.J., Strother R.M. (2015). Domperidone safety: A mini-review of the science of QT prolongation and clinical implications of recent global regulatory recommendations. N. Z. Med. J..

[27] Health Canada (2015). Domperidone Maleate—Association with Serious Abnormal Heart Rhythms and Sudden Death (Cardiac Arrest)—for Health Care Professionals.

[28] European Medicines Agency (2014). Domperidone-Containing Medicines. http://www.ema.europa.eu/ema/index.jsp?curl=pages/medicines/human/referrals/Domperidone-containing_medicines/human_referral_prac_000021.jsp&mid=WC0b01ac05805c516f.

